# Flagging the Fatty Acid Ferryman

**DOI:** 10.1371/journal.pbio.1002054

**Published:** 2015-02-03

**Authors:** Roland G. Roberts

**Affiliations:** Public Library of Science, Cambridge, United Kingdom

## Abstract

Fatty acids made in chloroplasts must be exported into the rest of the cell to be converted into commercially important plant oils. A new study identifies FAX1 as a protein that mediates this crucial transport step. Read the Research Article.

Oils from plant seeds provide the basis for many aspects of modern life that are taken for granted, being used to make cooking oil, soap, fuel, cosmetics, medicines, flooring, and many other everyday products. Whether derived from olives, oil palm, rapeseed, soybeans, peanuts, or sunflowers, the major constituents of these oils are triacylglycerides—glycerol molecules with fatty acids chemically connected to each of their three hydroxyl groups.

The fatty acids are where the bulk of the energy is invested, and where much of the value—for plants and humans—lies. In plants, these long, energy-rich molecules start life in the chloroplasts, where an ancient enzyme from these organelles’ bacterial legacy stitches two-carbon units together to make 16- or 18-carbon chains. When the growing chains reach this length, they are released and about 60% then exit the chloroplast for further processing in other parts of the cell, being further lengthened, edited and desaturated, or attached to glycerol to make triacyglycerides or membrane lipids (much of this happens in the endoplasmic reticulum).

While most of this process is well-known, the crucial mechanism by which the fatty acids get out of the chloroplast is less clear. We know that at the inner face of the chloroplast inner envelope membrane there is an enzyme that can clip the newly made fatty acid off the acyl carrier protein on which it has been built. We also know that at the outer face of the outer envelope membrane the free fatty acids become attached to the ubiquitous acyl-carrying molecule coenzyme A. Between these two covalently bound states in different compartments of the cell, however, the fatty acids, as free agents, must cross the chloroplast membranes. How they do so has been controversial; indeed, although there are some precedents for fatty acid transporters in other membranes, it is also thought that fatty acids could diffuse passively across membrane bilayers.

In a paper just published in *PLOS Biology*, Nannan Li, Katrin Philippar, and colleagues identify a novel protein involved in export of fatty acids from chloroplasts. The authors were studying a protein, FAX1, whose sequence suggested that it might be targeted to—and inserted into—chloroplast membranes. Although FAX1 proteins themselves are specific to green plants, they occupy one branch of a much larger protein family. Members of this family—the Tmem14 proteins—share a common structure of four α-helices, of which three cross the membrane; the fourth, which is amphipathic (oily on one side, polar on the other), may lie along the surface of the membrane. Although we don’t know much about what Tmem14 proteins do, they’re found across the eukaryote domain (and even in some bacteria); indeed, the structural information mentioned above was gleaned from human TMEM14A and TMEM14C.

The authors first confirmed that FAX1 is specifically inserted into the chloroplast inner envelope membrane in peas and the model plant *Arabidopsis thaliana*. To explore its function, they looked at two mutant *Arabidopsis* lines in which the *FAX1* gene is disrupted, and two in which it was overexpressed. The knockout plants were smaller than normal (Fig. 1), with near-empty seed pods (“siliques”), while the *FAX1*-overexpressing plants were significantly larger.

**Fig 1:**
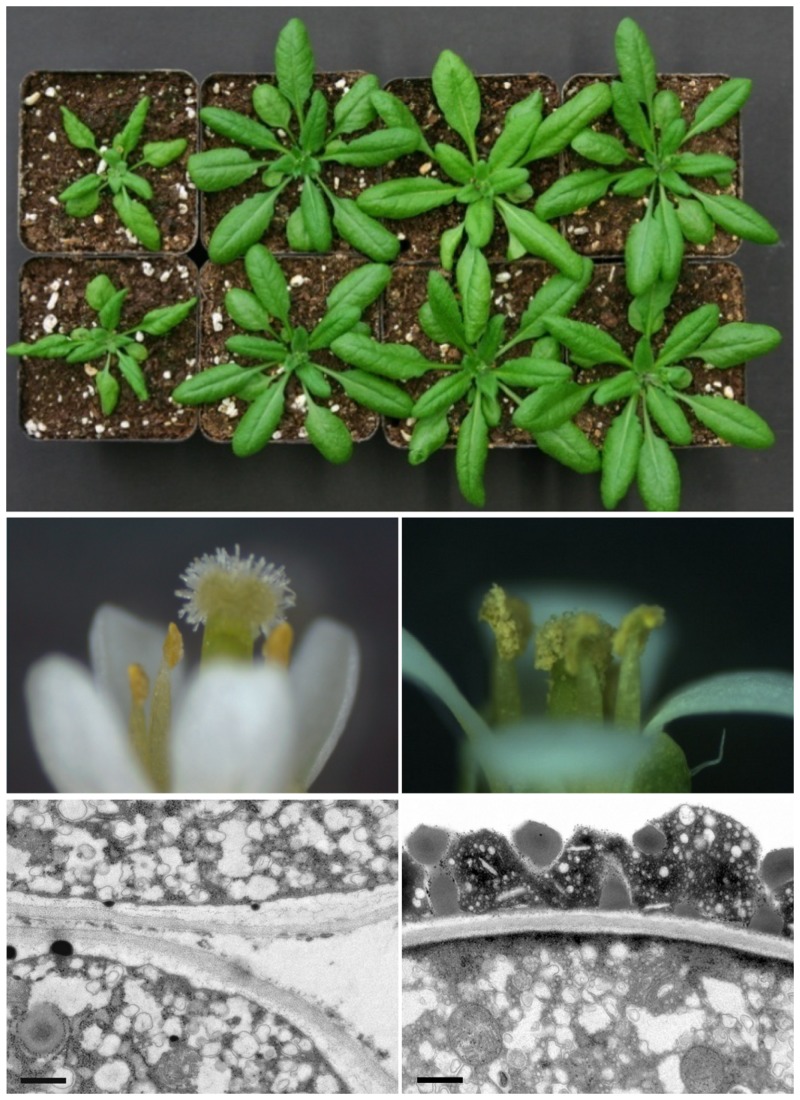
Without FAX1, *Arabidopsis thaliana* plants are small and flowers are male sterile because of “naked” pollen grains that lack the outer cell wall, which is made of fatty acid-rich components.

To pin down the cause of the striking effect on the siliques, the authors tested the fertility of the mutant plants, finding that the absence of FAX1 compromised the release of mutant pollen from anthers. Examination of the pollen showed that the normal yellow outer cell wall had failed to form, and the “naked” pollen grains remained lodged in the anther and thus became male sterile (Fig. 1). Notably, the complex chemicals that make up the outer layers of pollen (sporopollenin and tryphine) are known to be laid down by the tapetal cells in the anthers, using fatty acids as the primary starting material.

What about the rest of the plant? The size reduction of the knockout lines affected most parts of the plant, and the pronounced decrease in dry weight of the mutant plants (when compared to wet weight) pointed to a reduced amount of cell wall material. A closer look revealed a substantial reduction in cell wall thickness, including the outer layer of cutin and wax. The authors checked the composition of this layer, finding that the cutin was unchanged, but the amount of one ketone wax component was halved in *FAX1* mutant plants.

The combination of effects of loss of FAX1 on pollen cell wall and cuticular wax—both known to depend on plastid-derived fatty acids—suggested to the authors that the role of their protein in the chloroplast membrane might involve the export of fatty acids from this organelle. To explore this further, the authors surveyed the effects of FAX1 loss and overexpression on free fatty acids and fatty acid-containing lipids in leaves and flowers. The changes were complex, but the key findings are that, in total, 190 different fatty acid and lipid species (more than half those assessed) changed significantly, and that mutation tended to exert opposing effects on chloroplast-derived lipids and endoplasmic reticulum-derived lipids. Most strikingly, the triacylglycerides were strongly depleted in *FAX1* mutants (some by as much as 8-fold) and increased in FAX1-overexpressing plants.

Finally, the authors used mutant yeast as a test bed to directly assess the ability of FAX1 to mediate fatty acid transport. Yeast lacking the plasma membrane fatty acid transporter Fat1p are resistant to the presence of the toxic fatty acid α-linolenic acid in the culture medium, but if made to express FAX1 they succumb to the poison, presumably because FAX1 imports it. This effect does not happen with yeast lacking acyl-coenzyme A synthetases, implicating FAX1 specifically in transport rather than activation of fatty acids.

The authors combine all these data to assemble a strong circumstantial case for FAX1 being a fatty acid transporter in the chloroplast inner membrane. This is consistent with the protein’s peculiar transmembrane structure, with its sensitisation of yeast to externally supplied toxic fatty acid, with its contrasting effects on the distribution of lipids of chloroplast and endoplasmic reticulum origin, and with the striking effects of *FAX1* mutations on fatty acid-rich components of the waxy cuticle and pollen cell walls. In terms of mechanism, the authors speculate that the fatty acids might load onto FAX1’s amphipathic helix, causing it to dip into the membrane bilayer and flipping the fatty acid across. Most likely this is a passive transport process, driven by handover of fatty acids to coenzyme A on the cytosolic side, which lends directionality to export.

Given such a role for FAX1, what might other Tmem14 proteins do? The authors make the point that of the plant members of the family, three other (FAX2, FAX3, FAX4) are also expected to be targeted to the chloroplast, and their presence may account for the surprisingly mild phenotype of *FAX1* mutants. In nonphotosynthetic organisms like animals, Tmem14 proteins are candidates for the transport of fatty acids or related molecules across the membranes of mitochondria. As far as humans are concerned, given the commercial importance of plant oils, engineered up-regulation of FAX1 or its relatives (expression patterns suggest that FAX2, FAX3, and FAX4 may be more important for seed lipids), in combination with biosynthetic enzymes, might help boost oil yield for an increasingly demanding world.
